# Peracetic acid application as an antimicrobial and its residual (HEDP): a holistic approach on the technological characteristics of chicken meat

**DOI:** 10.1016/j.psj.2023.103003

**Published:** 2023-08-03

**Authors:** Heloísa Carneiro de Rezende, Marieli de Lima, Líbia Diniz Santos

**Affiliations:** Faculty of Chemical Engineering, Federal University of Uberlandia, Patos de Minas, MG, Brazil

**Keywords:** food safety, microbial intervention, poultry process, peracetic acid, HEDP

## Abstract

The most significant occurrence of food-borne diseases is due to *Campylobacter* and *Salmonella* contamination from chicken meat, and for this reason, strict regulations about strategies to improve the control of food pathogens are imposed by food safety authorities. Despite the efforts of poultry industry since the beginning of risk analysis and critical control point to reduce the burden of food-borne illness, technological barriers along the way are increasingly necessary to ensure safe food. The aim of this review was to carry out a scientific approach to the influence of peracetic acid (**PAA**) as an antimicrobial and its toxicological safety, in particular the stabilizer used in the formulation of PAA, 1-hydroxyethylidene 1,1-diphosphonic acid (**HEDP**), suggesting the possibility of researching the residual HEDP in meat, which would allow the approval of the PAA by the health authorities of several countries that still restrict it. This review also aims to ascertain the effectiveness of PAA, in different cuts and carcasses, by different application methods, comparing the effectiveness of this antimicrobial with other antimicrobials, and its exclusive or combined use, for the decontamination of poultry carcasses and raw parts. The literature results support the popularity of PAA as an effective intervention against pathogenic bacteria during poultry processing.

## INTRODUCTION

The public health impacts due to food-borne illness are enormous, in addition to significant social and economic burdens. Therefore, the safe production and distribution of food from field to table is crucial to ensure that consumers receive healthy food products. The World Health Organization (**WHO**) reports that annually, nearly 1 in 10 people worldwide fall ill after eating contaminated food, leading to more than 420,000 deaths. Children are disproportionately affected, accounting 125,000 deaths per year in people under the age of 5. Most of these cases are caused by diarrheal diseases. Other serious consequences of food-borne illnesses include kidney and liver failure, brain and nerve disorders, reactive arthritis, cancer, and death (WHO, 2022).

In this context, evaluating the U.S. history, a country that is efficient in food-borne illnesses reporting, the U.S. federal government estimates that there are about 48 million cases of food-borne diseases annually—the equivalent of 1 in 6 Americans becoming ill each year, these diseases result in 128,000 hospitalizations and 3,000 deaths annually. Although the American food supply is among the safest in the world, it reinforces the relevance of adopting procedures and control measures that corroborate with food safety (FDA, 2022).

In Brazil, 7,674 outbreaks were notified to the National Health Surveillance Agency (**ANVISA**) from 2009 to 2019, with 109 recorded deaths. The most commonly identified etiologic agents in ATD outbreaks were *Escherichia coli* bacteria, accounting for 29% of the total, followed by *Salmonella* and *Staphylococcus aureus*, with 17 and 16%, respectively (Maria et al., 2021).

Poultry slaughter and processing establishments regulated by official agencies must determine the “food safety hazards that may occur before, during, and after animals enter the establishment” through a farm-to-fork approach in their Hazard Analysis and Critical Control Point (**HACCP**) plan risk analysis. These interventions and procedures are necessary, as they are an integrated part of reducing the impact of *Salmonella* and other pathogens on public health. These pathogens are a danger that establishments producing raw poultry products must control using a HACCP plan or prevent in the processing environment employing a sanitary operational program (**HOP**), or other prerequisite program corroborating the HACCP plan for the control of carcass and poultry contamination by enteric pathogens such as *Salmonella* and *Campylobacter* spp. (Department of Agriculture - The Food Safety and Inspection Service, 2015; MAPA, 2017). In the same context, the European Union states that the main elements in poultry meat inspection are the accurate analysis of preslaughter batch information, antemortem examination of animals, postmortem examination of carcasses and organs, and programs that monitor the hygiene of the process. The control of these steps over the years can be seen by the reduction of *Campylobacter* counts in European and imported products compared to previous study periods. The last Annual Report of the Scientific Network on microbiological risk assessment, however, presented studies of resistant bacteria found within the poultry slaughter processes, such as *E. coli*. Out of the samples analyzed, 12.5% were tested positive, which is higher than expected in the literature (5–10%), reinforcing the constant challenge of the poultry segment to control pathogens (EFSA, 2021).

In 2019, the WHO reiterated the role of governments in treating food safety as a public health priority, as crucial play a key role in developing policy and regulatory frameworks and establishing and implementing effective food safety systems (WHO, 2022). Department of Agriculture - The Food Safety and Inspection Service (2015) reports that enteric contaminations are reasonably likely hazards to occur in poultry slaughterhouses, and that these should be controlled and monitored in the HACCP system through the critical control points of the establishment, and to identify described effective measures to reduce *Salmonella* and *Campylobacter* in raw poultry products.

For the United States Department of Agriculture, Food Safety and Inspection Service (**USDA-FSIS**), preventing food-borne diseases with the use of antimicrobial interventions in poultry meat is a priority for the industry. Concerned about meat safety, USDA-FSIS has developed a strategic plan for 2011 to 2016, formulating strict regulations and including goals that address strategies to improve food-borne pathogen control every year (Department of Agriculture - The Food Safety and Inspection Service, 2015; Kataria et al., 2020).

Although the American food supply is among the safest in the world, reinforcing the relevance of adopting procedures and control measures that corroborate with the safety of the food, the annual ATD report still brings worrying numbers of hospitalizations and deaths caused by these diseases (FDA, 2022). Therefore, the recommendation for use of microbial interventions is a possibility of adopting microbiological barriers for the control of alimentary pathogens (Department of Agriculture - The Food Safety and Inspection Service, 2015; Kataria et al., 2020). The Ministry of Agriculture and Supply in Brazil (**MAPA**) corroborates with the world inspection departments in the controls and risk evaluations of these diseases (MAPA, 2017). In terms of innovation in technological processes for the addition of barriers to microbiological control, Brazil, however, for regulatory reasons, is behind developed countries such as Canada, USA, Japan, and China and their regulations, that foresee the use of antimicrobial interventions, such as the use of peracetic acid (PAA), which has proven scientific basis as to its effectiveness for pathogen control. In the specific case of Brazil, the use of PAA as a coadjuvant technology in the function of antimicrobial agent in carcasses and/or parts of butcher's animals is approved by the Health Surveillance Agency - ANVISA, according to Resolution RDC No. 2 of January 8, 2004, in enough quantity to obtain the desired effect, without leaving residues in the final product (National Health Surveillance Agency, 2004). However, this approval does not provide for residues in the final product, and using coadjuvant technologies may result in the unintended but unavoidable presence of residues or derivatives in the final product (National Health Surveillance Agency, 2021). For this reason, the tolerance of the residual limits of PAA and its stabilizer (HEDP) concerning safety under the toxicological aspect, are requirements of the regulatory body of animal protein, enlightening the regulatory barrier in Brazil.

To progress on regulatory issues and achieve the food industry's goals for food safety, continued scientific investigations into progress on regulatory issues and achieve the food industries goals for food safety, and continued scientific investigations into microbial interventions in poultry processes are required. Research on final products that may be contaminated and that will be present on the consumer's table is a critical factor in improving the safety of poultry products and complying with legislation to achieve microbiological standards for *Salmonella* (Deumier, 2006; Scott et al., 2015; Cretu and Bondoc, 2022).

In the face of the mentioned, this paper demonstrates by reviewing literature studies, the efficacy and safety of the use of PAA as a technological barrier in poultry meat processes, and also evaluates the scientific impact regarding the research of this antimicrobial on sensory aspects and the residual of HEDP in raw poultry meat, as an incentive for the use of PAA in the prerequirement programs implemented in slaughterhouses.

## METHODOLOGY

Published and current national and international legislation and reference books regarding microbiological and technological aspects were consulted and served as a basis for the investigation to retrieve studies on the effect of the application of PAA.

Then, a web search containing scientific sources was conducted using the Science Direct, Web of Science, PubMed, and Springer platforms. The keywords used to retrieve the relevant information were: food safety, microbial intervention, poultry process, PAA and HEDP.

The survey covered the period from 2008 to 2021 and geographical restrictions were not imposed.

The search screening was based on the title, abstract and conclusion of the searched scientific articles. Articles classified as relevant met the criteria of i) providing relevant data and counts of bacteria for the study (*Enterobacteriaceae*, total mesophil count, *Salmonella*, and *Campylobacter*), ii) types of treatment, iii) comparison with other relevant antimicrobials, and iv) impact on the sensory and technological aspects of chicken meat by the action of antimicrobials.

After evaluating the selected documents, 2 standardized forms were designed to extract data from the selected articles. The first form aimed to collect general information from the studies and included information about the type of reference, the purpose of the study, where and when the study was conducted, and the kind of article.

The second form aimed to collect analytically relevant data for the scope of the review. It included information on the investigated bacteria, the analytical method used, the enumeration unit, data on the type of sample, application parameters of the antimicrobial solutions, kind of treatment, log reduction with the use of the antimicrobials, and the sensory aspects when cited in the articles. Only scientific publications that reported qualitative or quantitative microbiological reductions directly attributed to treatments using PAA, or its comparison with another intervention were used for data extraction.

The forms enabled the structuring of the bibliographic information and the detailing of this information as to the type of bacteria analyzed, the analysis methodology applied for the detection of the microorganism, the type of sample used (chicken cut or carcass), whether the microbiota was native or inoculated on a laboratory scale, the treatment applied with its controlled process parameters (immersion, spraying, chemical concentration, and application time), the log reduction after antimicrobial intervention and whether the citations addressed studies on the technological aspects of meat.

### Microbiota of Chicken Meat and Its Impact

Batz (2011) estimated in his study 14 food-borne pathogens that cause 14.1 billion (2,009 dollars) in disease costs annually and among these, 5 pathogens cause more than 90% of this health burden driven: *Salmonella* enterica, *Campylobacter* spp., *Listeria monocytogenes, Toxoplasma gondii*, and Norovirus. Among these 14 pathogens found in food overall, a ranking of societal impact and risk was performed, using as criteria disease costs, number of patients, hospitalizations, and deaths. *Salmonella* is the first pathogen of relevance and is the biggest cause of food-borne diseases than any other pathogen, causing high numbers of hospitalizations and deaths. In a second place is *Toxoplasma gondii* and in third place is the pathogen *Campylobacter*.

Since *Salmonella* is the primary pathogen of relevance, foods involved in salmonellosis outbreaks are associated with a wide variety of foods regulated by FSIS and FDA, with significant risks associated with poultry, poultry products, and eggs (WHO, 2022). This suggests that the reduction of the *Salmonella* load requires an enormous effort from the regulatory agencies as well as from the slaughterhouses in order to guarantee process control and consequently reduce the risk of cross-contamination in the poultry process (Department of Agriculture - The Food Safety and Inspection Service, 2015).

Contaminated poultry has the greatest public health impact among foods. The most significant disease relevance is due to contamination with *Salmonella* and *Campylobacter* which are among the top 3 in the ranking of the top 10 food-borne pathogens of public health concern(Batz, 2011; Forsythe, 2013; WHO, 2022).

**Figure 1:**
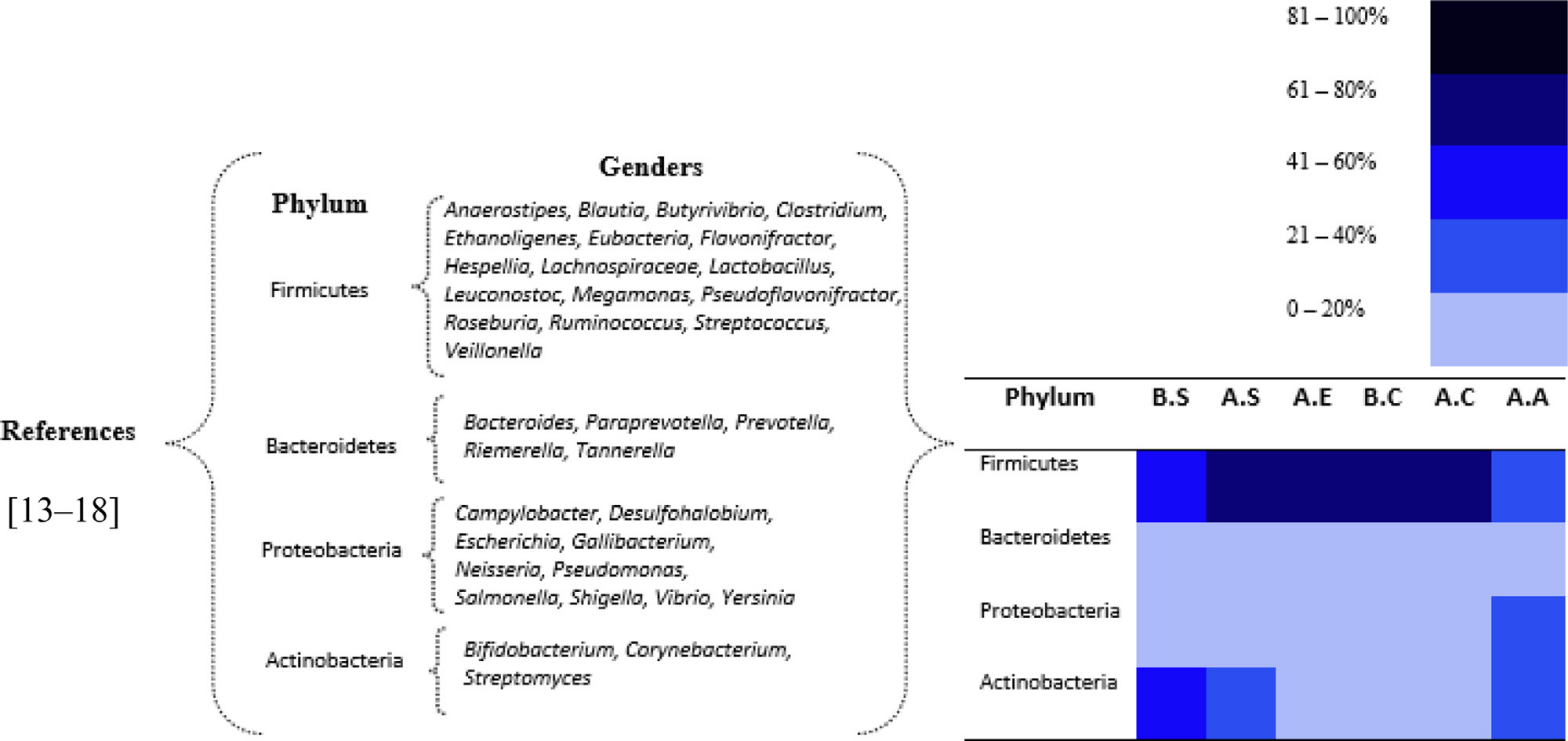
Relative taxonomic unit abundance at phylum level in 6 sample groups across the poultry processing line. Where: BS group: samples collected before scalding; AS group: samples collected after scalding; AE group: samples collected before evisceration; BC group: samples collected before immersion cooling (chiller); AC group: samples collected after immersion cooling (chiller); AA group: postair cooling samples.

Understanding the bacterial community profile through poultry processing can help the industry to know itself and consequently produce better poultry products. A study by Chen et al. (2020) evaluated the most abundant phyla found in chicken carcasses in the production process. In general, 98.2% of the organisms belonged to the phyla Firmicutes, Proteobacteria, Bacteroidetes, and Actinobacteria as schematically shown in Figure 1.

When comparing the relative abundance of phylum between the beginning of processing (the BS sample group) and the end of processing (the AA sample group), it can be seen that the only phylum that became more predominant was the Proteobacteria phylum, as it increased its relative abundance over the course of the process. This phylum increased 32-fold in relative abundance from 0.9% in the sample group before BS evisceration to 28.8% in the sample group after AA air cooling (last step of the process). This can be explained by the prevalence of 2 pathogens within the phylum, that is, *Salmonella* and *Campylobacter*. The study in question also evaluated the prevalence of these pathogens and their presence was observed in all the stages of the production process evaluated with a prevalence of 89% for *Campylobacter* and 20% for *Salmonella* in the analyzed chicken carcass samples (Chen et al., 2020).

### Chicken Slaughter and Contamination Points

From the second half of the twentieth century to the present day, agribusiness in Brazil has achieved significant advances in order to compete globally with the major world powers, enabling technological advancement of poultry production which is impressive for its dynamism and competence achieved in recent decades (Brazilian Animal Protein Association, 2022).

Plants processing large quantities of chicken increasingly need hygienic practices during processing. It is essential to ensure food safety during slaughter, to control the spread of stool and cecal content, as these are the main hosts of enteric pathogens such as *Salmonella, E. coli*, and *Campylobacter* (Demirok et al., 2013; Projahn et al., 2019).

**Figure 2:**
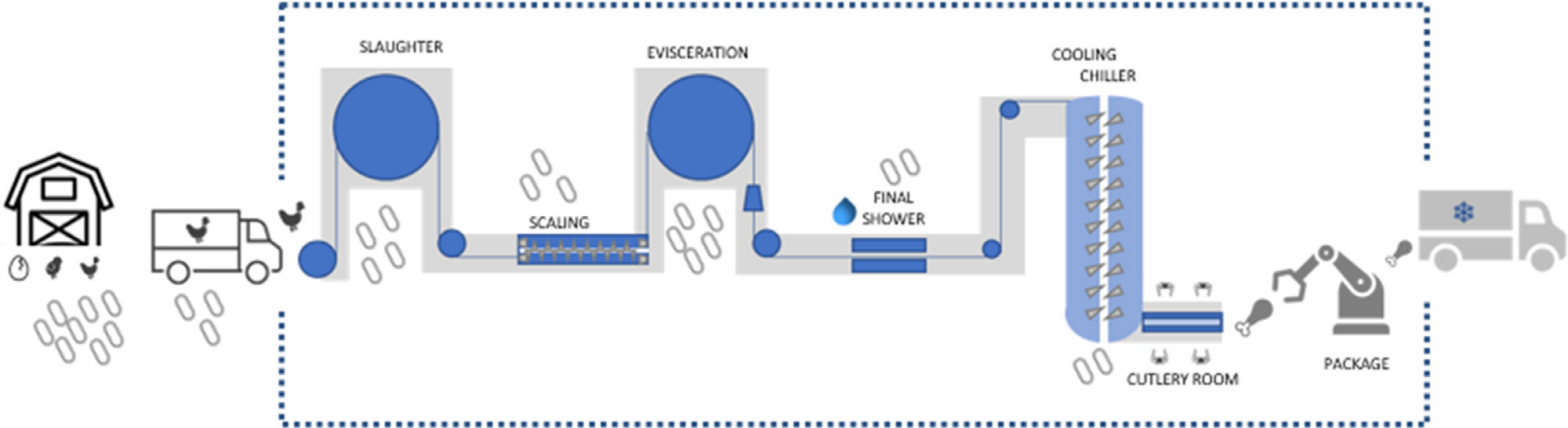
An overview of the poultry meat production chain (farm-to-fridge), indicating the main processing steps and the prevalence of bacteria present in the poultry microbiota at each step.

Based on the routine of a slaughterhouse in the mining region of Brazil, the poultry slaughter processing is detailed according Figure 2.

### Reception and Hanging

Approximately 24 h before slaughter, the feed is removed and the chicken will be fasted. Properly performed preslaughter fasting aims to reduce the amount of fecal material in the animal's gastrointestinal contents. This prevents defecation during transport and the associated microbial spread among the chickens in the transport cases on the way to slaughter. This step is crucial to ensure the process of efficient evisceration, a prolonged fasting time causes changes in the animal's metabolism, friable viscera, and consequent intestinal rupture in the eviscerator, causing cross-contamination (Foley et al., 2011; García-Sánchez et al., 2017; Marmion et al., 2021).

The transportation of the birds from the poultry farm to the slaughterhouse is done by special trucks, equipped with specific cages to transport them. In these cages, the quantity of birds housed depends on the average weight of the bird, but always guarantees the animal's well-being.

On arrival at the company, the Federal Inspection visually inspect the birds in the lot to be slaughtered. After this procedure, the birds, still in the truck, are housed in an area with fans and water sprinklers, to ensure rest and reduce stress during transport, with controlled temperature and humidity (Bondoc, 2014; MAPA, 2017).

### Slaughter

The stunning and bleeding room, where the bird arrives after being hung, should have low light intensity and be away from loud noises to reduce stress to the animal. In this area stunning by electronarcosis is performed. The stunning tanks contain water, with a flow rate dependent on the volume of birds, and the frequency, amperage, and voltage are standardized. The stunning time is a maximum of 12 s (Ministry of Agriculture, Livestock and Supply, 1998).

The birds are bled manually with a knife, which aims to cut the carotid arteries and jugular veins. The knives are replaced every 30 min and sterilized at a minimum temperature of 85°C. After this process, the bird spends at least 3 min in a bleeding tunnel, so most of the blood can drain away (Ministry of Agriculture, Livestock and Supply, 1998).

A study has evaluated the prevalence of enteric *Salmonella* in slaughter processing equipment after complete sanitization procedures, and found that *Salmonella* persisted significantly in the first equipment of the slaughter line (Obe et al., 2020).

The study in focus, corroborates the fact that slaughter and bleeding are documented points at which about 60% of carcasses can carry the highest *Salmonella* cell burden, about 6.1 log CFU/ g, found during the processing chain (Rivera-Pérez et al., 2014; Boubendir et al., 2021). This can be explained by the high contamination of feces, dirt, and feathers that are inherent to the birds, as well as possible stress factors that lead to changes in the bird's metabolism, from fasting until the moment of slaughter.

### Scalding and Plucking

Scalding is done by immersing the bird in tanks containing water at a controlled flow rate and temperature, and the water is heated by steam. The water temperature in the scalding tank is between 54°C and 63°C. The birds go through this process to physically remove dirt from the skin and feathers and mainly to open and soften the feather follicles, facilitating their removal in the plucking machines (MAPA, 2017).

The scalding process is the first critical control point in poultry processing, since the step can result in the initial attachment of *Salmonella* to the skin. These *Salmonella* contaminated carcasses can result in cross-contamination with the other carcasses via the scalding water and transporting the contamination throughout the subsequent stages of processing (Mcbride et al., 1980). Maintaining a constant renewal of the water in the scalding tank reduces the possibility of cross-contamination in the step, as this is the first viable point for the application of antimicrobial agents by the renewal water, helping to reduce microbial counts (Chen et al., 2020).

Then the carcasses go through the plucking machines to remove the feathers using rubber fingers and water heated by steam at a temperature between 60°C and 70°C. These machines are regulated for each size and average batch weight, slope, height, and finger hardness. Maintenance must occur frequently to avoid carcass fractures, scratches, torn skin, and bruises that compromise the quality of the final product. After plucking, the carcasses go through the initial shower with controlled flow to remove possible dirt and feathers.

Washing can occur at various stages of the production cycle, and depending on the regulations of each country, chemical antimicrobials can be used as a technological barrier in microbiological control (Buess et al., 2019; Boubendir et al., 2021; Gomes da Silva et al., 2021).

### Evisceration

The evisceration process begins with the preinspection conducted by the Federal Inspection (**FI**). The carcasses go through the process of opening the abdomen and exposing the viscera. Then, at the exit of the eviscerator, the carcasses have their packages of offals aligned with the respective carcass so that the FI can also inspect the package of offals. After this process, the carcasses are released and proceed to the crop and trachea extractor and neck extractor (MAPA, 2017).

The carcasses then proceed to the monitoring stage, where the carcasses that still have visible gastrointestinal contamination are removed. Then, they go through the final shower with controlled flow, to remove possible residues that still exist, and proceed to the water immersion cooling system (*chiller*) (Ministry of Agriculture, Livestock and Supply, 1998).

The evisceration stage is the most sensitive stage in controlling the spread of contamination. This is because it is the time when all the bird's intestinal contents are exposed. This process, either manually or mechanically, can cause ruptures in the cecal coating. In addition, the high load (about 11 log CFU/g) of bacteria per gram of cecal contents provides a strong potential of contamination if exposed (Waite and Taylor, 2014). For this reason, the operational sanitary controls evaluating the efficiency of the equipment alongside the fulfillment of the preslaughter fasting procedure, help in the control of the discrimination of the contamination in the evisceration stage (Rivera-Pérez et al., 2014).

In addition, equipment and surfaces are particularly vulnerable to cross-contamination of carcasses in this part of the plant. A study conducted in France recovered *Campylobacter jejuni* from the eviscerator before cleaning and disinfection, and this pathogen persisted in the equipment and was recovered again after the disinfection procedure, which corroborates the relevance of preslaughter procedures to reduce contamination within slaughterhouses (Peyrat et al., 2008; Obe et al., 2020).

A strategy adopted to reduce the microbial count throughout the process is the application of antimicrobials at this stage, through the initial washing showers (before evisceration) and the final shower, at the end of the evisceration process (EFSA, 2008).

### Carcass Cooling (Chiller)

Precooling of carcasses is done using chillers, which transport the carcasses through 3 cooling stages, making the bird reach a maximum temperature of 4°C. The chilled water enters countercurrent at a flow rate according to the carcass weight. In the first stage, called prechiller, the residence time should not be longer than 30 min and the cooling and washing of the carcasses begin, the water temperature inside this chiller has a maximum limit of 16°C (Ministry of Agriculture, Livestock and Supply, 1998).

The second and third stages, like the first, will lower the carcass temperature, and the water temperature should be a maximum of 4°C. The retention time of the carcasses in these 2 chillers is approximately 120 min. After the cooling period, which takes about 2 h, the carcasses leave the chiller with a maximum of 4°C and are sent for rehanging. At this stage the carcasses are rehung and directed to the dismembering and deboning area. The period from rehanging to the next area should ensure a satisfactory drip.

Effective cooling can reduce bacterial levels that may have been elevated during the previous stages, especially plucking and evisceration, paying special attention to the pathogens of relevance in the poultry process, *Salmonella, E. coli*, and *Campylobacter*, as they exhibit high thermosensitivity at cold temperatures (Hardie et al., 2019; Boubendir et al., 2021). Its effect, however, depends on factors such as initial microbial load, chemical additions to the cooling system, water flow rate, and cooling capacity (Demirok et al., 2013; Ae Kim et al., 2017; Marmion et al., 2021).

### Cutting Area

Leaving the chiller, the carcasses go through the conveyor to the cutting sector. In the cutting area, where the ambient temperature is a maximum of 12°C, the cuts are completely removed, where the bone is removed and the skin may or may not be removed and proceeds to the packaging process (Ministry of Agriculture, Livestock and Supply, 1998).

Afterward, the cuts are weighed and packed in printed plastic packaging (separately by type of product), then they go to the freezing tunnel, which operates at a temperature between −25°C or colder, until the product reaches a minimum temperature −12°C for the internal market and −18°C for the external market, being ready to be shipped (Ministry of Agriculture, Livestock and Supply, 1998). Storage is an important step to a refrigeration chain that prevents the accelerated growth of microorganisms and thus can extend the shelf life of meat (Cretu et al., 2016).

In this stage of the process, the immersion of cuts after the cooling system was presented as the most used in the published literature, this corroborates ensuring the safety of the final product (Bourassa et al., 2019; Cano et al., 2021).

### Organic Acids Used in Pathogen Control

When selecting an antimicrobial intervention, establishments should ensure that the interventions and levels of antimicrobials used are safe and appropriate, demonstrated by the Table 1.

**Table 1:** Summary of updates to list of substances used as an antimicrobial intervention.

Substance	Amount
Watery solution of citric and hydrochloric acids	Sufficient for purpose
Lactic acid	Solutions of 2–5% lactic acid and a minimum 2:1 ratio of lactic acid to sodium lactate
Watery mixture of sodium diacetate, lactic acid, nisin preparation, and pectin	Not to exceed a 20% solution of the aqueous mixture, and not to exceed a 0.2% nisin concentration
Aqueous mixture of peracetic acid (PAA), hydrogen peroxide (HP), acetic acid (AA) and (HEDP), optionally sulfuric acid (SA)	2,000 ppm PAA, 1,474 ppm HP, and 136 ppm HEDP
Aqueous mixture of peracetic acid and xanthan gum	Concentration not to exceed 1,500 ppm PAA, 800 ppm HP, 133 ppm HEDP, and 0.5% xanthan gum
Bacteriophage preparation of up to 6 phages targeting *Salmonella*	up to a level of 1 × 10^8^ CFU/g of food
Aqueous solution of citric and hydrochloric acids adjusted to a pH of 1.0–2.0	Sufficient for purpose
Aqueous solution of citric and hydrochloric acids adjusted to a pH of 0.5–2.0	Sufficient for purpose
Aqueous solution of sulfuric acid and sodium sulfate	Concentration sufficient to achieve a targeted pH range of 1–2.2; delivered at a minimum system pressure of 0.5 psi

United States Department of Agriculture Food Safety and Inspection Service.

To this end, Directive 7120.1 (Department of Agriculture - The Food Safety and Inspection Service, 2021) regulates the use of antimicrobial agents from safe and suitable ingredients in the production of meat, poultry, and eggs.

However, just the Directive 7120.1 is not enough scientific support for the use of interventions by establishments, because it does not contain effectiveness data or all the critical operational parameters. Thus, the scientific opinions issued by the health authorities of each country are extremely relevant for determining operational criteria, since the opinions issued by the health authorities are based on several studies and evidence.

Antimicrobial agents can be used at 3 stages in poultry processing: i) on hot eviscerated carcasses or parts (precooling) by short-term spray or immersion; ii) on carcasses in the chiller system; iii) on chilled carcasses or parts (postcooling) by short-term immersion. Usually the antimicrobial is added to the water in equipment already present in the processing line (European Food Safety Authority, 2014a).

Ebel et al. (2019) have researched 167 poultry slaughterhouses in the United States that produce chicken carcasses and cuts and identified that most establishments use PAA as the main antimicrobial intervention for both carcass application (immersion and spray) and immersion cuts.

Sukumaran et al. (2015) have studied the combination of organic acids (PAA, chlorine, cetylpyridinium chloride, and lauric arginate) with phages. In their study, the best combination for *Salmonella* reduction was the use of 400 ppm PAA per 20 s of chicken skin immersion, enabling a reduction of up to 2.5 log CFU/g. In the same study, the efficacy of using only PAA was observed, achieving a reduction of up to 1.7 log CFU/g of the pathogen, a result greater than the combination of the use of phages with the other organic acids.

Furthermore, the results of several studies over the past 7 yr suggest the use of PAA as an effective antimicrobial strategy for application in slaughterhouses to reduce *Salmonella* and *Campylobacter* in carcasses, corroborating the popularity of PAA (Nagel et al., 2013; Barco et al., 2015; Scott et al., 2015; Sukumaran et al., 2015; Ae Kim et al., 2017; Walsh et al., 2018; Zhang et al., 2019; Cano et al., 2021).

PAA, also known as PAA or peroxyacid, is a mixture of PAA, acetic acid, hydrogen peroxide, and 1-hydroxyethylidene-1,1-diphosphonic acid (**HEDP**), optionally octanoic acid, and sulfuric acid. Its use in raw poultry products has been approved by the Food and Drug Administration (**FDA**) at a maximum concentration of 2,000 ppm peroxyacids (Department of Agriculture - The Food Safety and Inspection Service, 2021).

### Regulatory Framework in the Evaluation of the Safety and Efficacy of Peracetic Acid Solutions

PAA solutions are produced from water, acetic acid, hydrogen peroxide (**HP**), and HEDP. Alternatively, octanoic acid and sulfuric acid can be used. PAA formation is the result of an equilibrium reaction between HP and acetic acid. Although acetic acid and hydrogen peroxide are known to have antimicrobial properties, their effects within these solutions are minimal. Acetic acid reacts with hydrogen peroxide to generate PAA with which it is in equilibrium (European Food Safety Authority, 2014a; Bertram et al., 2019).

Hence, the amount and presence of acetic acid and hydrogen peroxide are critical to the concentration of PAA and thus the antimicrobial effect. HEDP has no antimicrobial effect, it functions as a stabilizer in the solution, preventing metal ions from catalyzing the degradation of PAA and hydrogen peroxide (Joint FAO/WHO Expert Committee on Food Additives, 2006).

Acetic acid is oxidized by hydrogen peroxide, resulting in PAA and water, this reaction tends to equilibrium. The percentage of conversion of acetic acid into PAA depends on the relative molar ratio between the reactants, their concentrations and the presence of the acid catalyst. Generally based on formulated PAA, it uses the substance of HEDP as a catalyst and stabilizer of the formulation. It is known that PAA solutions are considered stable, as well as the concentrated product. The most common market concentrations range from 10 to 15%, mainly due to reactivity and instability at higher concentrations. Studies on the kinetics of synthesis and decomposition of PAA show that the use of substances with sequestering characteristics, such as HEDP, increases the shelf life of concentrated products by approximately 1 yr after manufacture (Joint FAO/WHO Expert Committee on Food Additives, 2006). Zhang et al. (2017) in his study on thermodynamic properties of an emerging chemical disinfectant, PAA reports that oxidizing acetic acid with hydrogen peroxide in the presence of a catalyst such as sulfuric acid is a common approach for PAA synthesis or perhydrolysis.

The maximum use concentration of PAA, hydrogen peroxide, and HEDP for application in poultry products is described in Table 2.

**Table 2:** Allowed concentration of PAA, H_2_O_2_, and HEDP solutions in industrial poultry product operations.

Intended use	PAA (ppm)	H_2_O_2_ (ppm)	HEDP (ppm)
Water used in processing, ice or brine, spraying, immersion, rinsing, cooling system, low temperature immersion baths (below 4.4°C), or scalding water for whole or cut poultry, including carcasses, parts and organs	2000	1474	136

The Joint FAO/WHO Expert Committee on Food Additives (**JECFA**) estimated the intake of each component of the PAA solution based on the residual amounts expected to be present in the treated food (meat and vegetables) at the time of consumption (Joint FAO/WHO Expert Committee on Food Additives, 2006).

The ingestion of acetic acid is not a concern, since its use as vinegar on food would result in a much higher exposure than using peracetic antimicrobial solutions (European Food Safety Authority, 2014b).

JECFA indicates in its conclusion that because the peroxide compounds reactivity, only octanoic acid, acetic acid, and HEDP remained in foods that are treated with the antimicrobial solution and that are not subsequently washed, processed or cooked (Joint FAO/WHO Expert Committee on Food Additives, 2006).

The EFSA assessment of 2005 cites experiments performed to establish residues of PAA, peroxyoctanoic acid, and HEDP (EFSA, 2005). In these experiments, the residues of peroxyacids and hydrogen peroxide in chicken carcasses after 2, 5, and 10 min of peroxyacid spraying (200 mg/L) and immersion for 60 min at below 4°C were below the detection limit of 1 mg/L. Because of the low levels of peroxide compounds observed and the chemical instability/reactivity, it is unlikely that these substances will remain on the poultry carcasses and therefore there is no need to perform a safety assessment for these substances. Regarding HEDP, 6 chicken carcasses were treated with 2 different solutions. Solution 1 contained 200 mg/L peroxyacids (as PAA) and 10 mg/L HEDP, and solution 2 contained 30 mg / L peroxyacids and 1.5 mg/L HEDP. All chicken carcasses were sprayed for 15 s with solution 1 at room temperature. Three of the chicken carcasses were then immersed for 60 min in a 3°C bath with solution 1, and the other 3 chicken carcasses were immersed for 60 min in a 2°C bath with solution 2. Chicken carcasses treated with solution 1 in the bath gave a residual amount of 120 to 170 μg HEDP per kg carcass. In the case of solution 2 in the bath, the residual amount was 40 to 50 μg HEDP per kg carcass, close to detection limit (**LOD**).

Based on the previous information, it can be seen that HEDP can remain in the carcasses after treatment, which are not subjected to further washing or processing.

According to the JECFA risk assessment, several studies (human, rat, rabbit, dog, and monkey) have been conducted on the elimination of HEDP after oral administration. Collectively, the data indicated that the absorption of HEDP from the gastrointestinal tract is quite limited and that its metabolism is insignificant. Some accumulation in bone has been observed, with a half-life in rats of about 12 d (Joint FAO/WHO Expert Committee on Food Additives, 2006).

HEDP did not induce mutations in a bacterial gene mutation assay (Ames test) nor in an in vitro mammalian cell gene mutation test in L5178Y TK ± mouse lymphoma cells (Joint FAO/WHO Expert Committee on Food Additives, 2006).

It is worth noting that studies related to HEDP absorption in humans and animals corroborate the safety of the stabilizer with respect to immersion bath at high concentrations. This conclusion, though, is only applicable for PAA working solutions containing up to 130 mg HEDP/L in combination with 3-min immersion times. When longer contact times are applied, EFSA recommends that HEDP concentrations should be correspondingly reduced. Furthermore, as already mentioned, for long duration baths, HEDP residues remain on carcasses after microbial treatment (European Food Safety Authority, 2014a).

Various amino acids and amino acid derivative compounds, such as peptides and proteins, can be oxidized by the peroxyacids present in the PAA solution (EFSA, 2005). Although there are several possibilities for amino acid oxidation, it was concluded that “no significant level of amino acid by-products will be produced after peroxyacid treatment, since the levels of free amino acids in chicken meat just before aging are very low” (EFSA, 2008). The application of peroxyacids solution can also cause oxidation of fatty acid lipids with one or more double bonds (EFSA, 2008). In this regard, in 2005, EFSA concluded that no significant differences were observed in thiobarbituric acid reactive substances (**TBARS**) values or fatty acid profiles when comparing treated samples with raw or cooked samples (EFSA, 2005).

Both the risk assessments conducted by JECFA and EFSA conclude that the use of the evaluated solutions is not of health concern.

The scientific opinion on the evaluation of the safety and efficacy of PAA solutions for pathogen reduction in poultry carcasses and meat (European Food Safety Authority, 2014a) concludes that no toxicity concerns have been identified regarding peroxyacid residues due to the high instability described. No concerns are indicated, either, regarding residual acetic acid and octanoic acid.

Moreover, concerning the question of the safety of possible reaction products of hydrogen peroxide and peroxyacids with lipids and proteins/amino acids from poultry carcasses, due to the low content of amino acids on the carcass surface it was concluded that no risk was expected, including short-term treatment with higher peroxide concentrations (European Food Safety Authority, 2014a).

In regards to lipid peroxidation, no by-products were identified in the experiments present in the EFSA, 2005 risk assessment (EFSA, 2005, 2014a).

Given the aforementioned, regarding the safety and efficacy of PAA solutions, European Food Safety Authority (2014a) corroborates with the safety of PAA, however, as far as the stabilizer HEDP is concerned, with regard to long-term immersion baths, it is recommended to control HEDP residues in poultry carcasses and the monitoring of HEDP concentration in the PAA working solution should be considered in HACCP plans. The recommendations provided in the scientific opinion of the European Food Safety Authority support the risk assessment of the HEDP stabilizer based on the mentioned studies.

### 1-Hydroxyethylidene 1,1-Disphosphonic Acid Stabilizer

HEDP belongs to a group of synthetic chelating agents, which are widely used for various applications, for example, as corrosion inhibitors, antioxidants, antifouling agents, dispersants in cooling water circulations, and as building blocks in industrial cleaning products and detergents. Due to its ability to slow or prevent the precipitation of minerals, especially Ca salts from aqueous solutions at substoichiometric concentrations (threshold effect), HEDP has found preferential application in the area of water conditioning and water softening, partially in combination with zeolite (Fischer, 1993).

As recommended by European Food Safety Authority (2014a) a method for the determination of HEDP residues in poultry carcasses, poultry meat and poultry meat products should be developed and validated.

**Figure 3:**
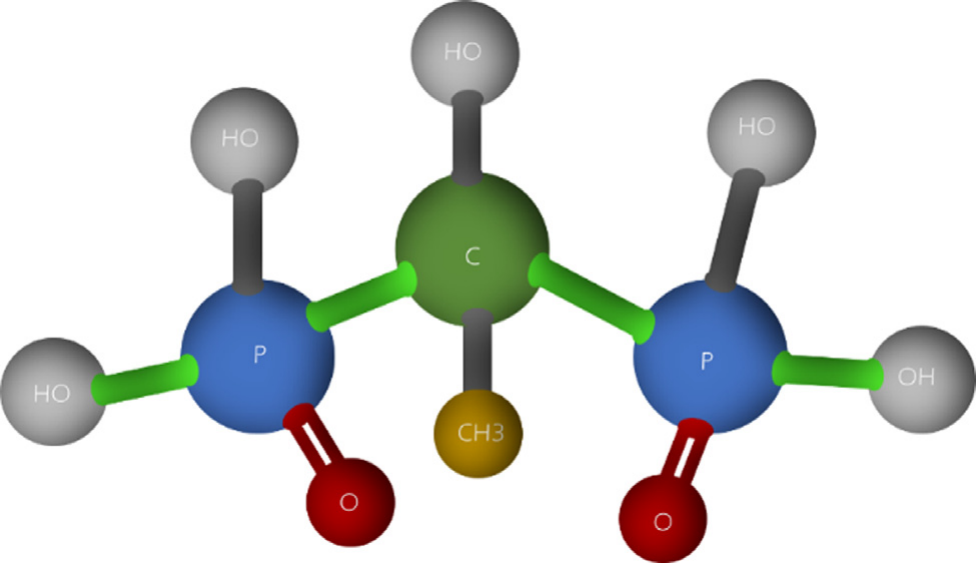
Molecular structure HEDP-C_2_H_8_O_7_P_2_.

Figure 3 demonstrates the minimum molecular structure of the HEDP molecule defined as C_2_H_8_O_7_P_2_. By basic stoichiometry, each molecule of HEDP has 2 moles of phosphorus, that is, phosphorus works as an indicator of the presence of HEDP, because in the other raw materials used in the composition of PAA, there is no phosphorus present.

To understand the mechanism of HEDP as a stabilizer for PAA, it is important to note that PAA dissociates rapidly into water and acetic acid, especially upon contact with proteins (Gamble et al., 2016; Walsh et al., 2018). PAA provides only a large initial reduction in bacteria due to the organic material containing nitrogen in the muscle. After 1 min of contact time, there is no measurable PAA residue remaining and therefore HEDP is an additive present in the peracetic composition to prevent precipitations in chemical reactions and allow it to happen (Joint FAO/WHO Expert Committee on Food Additives, 2006; Mesrobian, 2009).

Since HEDP is essential to stabilize the reaction and according to EFSA's study that ensures that HEDP residues remain on carcasses after microbial treatment for long duration immersions, the search for HEDP residues in meat matrix is relevant in cold storage processes, thus ensuring the LOD (European Food Safety Authority, 2014a).

### Impact of Using Organic Acids on Meat Technological Aspects

It is known that one of the significant factors influencing consumer preference for chicken meat is the meat color at the point of purchase, an important attribute in the appearance evaluation. Color preferences are a subjective characteristic of meat, as perceived by the consumer. Consumers tend to prefer chicken meat that is very similar in color to what they are used to consume (Manjankattil et al., 2021).

To measure, classify and reproduce color readings, it was necessary to develop objective instrumental parameters. The food color is most often measured in terms of the CIE scale, where the L*, a*, b* values plus the shade angle and chroma are quantified. The CIE Lab color space defines the L*, a*, and b* values in relation to an international color standard measurement, adopted by the Commission International Eclairage (**CIE**) in 1976. In this method, the L* value is the brightness component, ranging from 0 to 100 (black to white); a* and b* range from −120 to +120 with a* ranging from green if it is negative to red if it is positive and b* ranging from blue if it is negative to yellow if it is positive (Yam and Papadakis, 2004; An et al., 2010). The poultry meat color is instrumentally measured by means of a colorimeter that measures the CIE values L*, a*, and b*; the colorimeters only measure a meat area between 2 and 5 cm^2^ (Manjankattil et al., 2021).

Studies evaluating the color change in chicken legs treated with lactic acid were not satisfactory when compared to the control sample. Experiments conducted between 2003 and 2017 on red meat cuts treated with lactic acid by immersion with concentration of 2, 4, and 6%, found that meat treated with lactic acid had higher L* values compared to untreated meat (Wideman et al., 2016; Manzoor et al., 2020).

Studies that addressed color analysis in chicken meat treated with malic acid, benzoic acid both with concentration ranged from 0.1 mg/mL to 5 mg/mL and citric acid (2%), found that these acids did not cause significant changes when compared to control samples (Wideman et al., 2016).

The texture, mainly of chicken breast, is a relevant meat characteristic in the definition of product quality by the consumer. Antimicrobial treatments can affect these characteristics (Wideman et al., 2016) because they generally have a low pH, which can cause a sharp decrease in pH after slaughter. If the pH value decreases rapidly right after slaughter, it can cause alterations in the muscle structure, resulting in pale, flaccid meat with low water retention capacity, which is then called pale, soft, exudative (**PSE**). For instance, PAA has a very low pH (2.6–3.4) and it is essential to study its reaction on the sensory aspect of meat (Kataria et al., 2020).

Aside texture and color technological characteristics are also cooking loss, water-holding capacity, and drip loss, which are influenced by pH that could change due to the application of PAA.

For this reason, studies that corroborate the identification or not of sensory alterations in meats submitted to antimicrobial treatment are necessary.

Among the most widely used techniques to evaluate the meat resistance to mechanical and crumbling effects are those that apply shear force, which measures the force required to cut food, using universal texturometers (Lyon and Lyon, 1998; An et al., 2010). Another study to evaluate the identity and quality of products with great applicability is the sensory analysis by trained judges, through the discriminative control difference test. It determines the probability of difference or similarity between 2 products, that is, if there is a significant difference between the test and control (untreated) samples, and estimates their degree of difference (Skelton, 1984). Thus, evaluating the characteristics of chicken meat through the objective analysis shear force is a unique strategy to evaluate possible changes in meat texture caused by the use of antimicrobials, which would impact consumer preference and acceptability.

Regarding the technological effects, Nagel et al. (2013) evaluated the sensory attributes in chicken (breast) meat treated with antimicrobial solutions containing PAA, including appearance, flavor, texture, juiciness, and general acceptance after treatment and after 24 h of storage at +4°C. There were no significant differences (*P* > 0.05) between chicken breast treated with 400 ppm and 1,000 ppm PAA solution in immersion in postchiller system when compared to the control sample considering the appearance, flavor, juiciness, and overall acceptability attributes. The chicken meat appearance after all treatments was rated as “slightly equal.” Similarly, the panelists were unable to determine differences between the various treatments for the flavor attribute.

The treatment with 1,000 ppm PAA was rated as “moderately tender,” while the treatment with 400 ppm was identified as “slightly tender” for the texture sensory evaluation. When the participants evaluated the juiciness of the breast fillets, none of the treatments showed significant differences (*P* ≤ 0.05) from the control sample and were perceived as “slightly moist.” Bauermeister et al. (2008) also evaluated sensory aspects and in their study, the application of PAA in different concentrations did not differ significantly from the control (*P* > 0.05).

Manjankattil et al. (2021) evaluated characteristic of meat regarding color and in his study, the color evaluation parameters L*, a*, b* did not differ significantly between the control sample and the sample treated with PAA (*P* > 0.05).

It is relevant to highlight that among the articles selected for the review, only the aforementioned authors approached the study regarding the sensory aspects of meat; however, 2 of them approached consumer perception through sensory analysis and only 1 brought the objective analysis of color.

Extensive research was carried out and no articles were found that addressed the objective meat characteristics of tenderness, cooking loss, water-holding capacity, and drip loss. It is known that the reduction in pH can directly influence the meat quality, thus studies that address it are relevant.

Bertram et al. (2019) evaluated a spraying treatment, *C. jejuni* (10^8^ CFU/mL) inoculated chicken drumsticks and native skin-on breast fillets were treated for 30 s with PAA of 1,200 ppm concentration. Samples were packaged and stored at 4°C until further analysis. On d 1, 6, and 12, the fillets were used for pH analysis. No significant differences of the pH results could be observed on all storage days between the untreated, water-treated, and PAA-treated breast results could be observed on all storage days between the untreated, water-treated, and PAA-treated breast fillets after application.

Park et al. (2017) also found no differences of the pH values in ground chicken after dipping the breast skin in 1,200 ppm PAA.

The above studies demonstrate that the PAA action may not be able to change the isoelectric point of the meat, which would consequently change the pH and possibly the technological characteristics. A possible explanation is that is a superficial decontamination, where the cuts are treated in high concentrations but for an extremely fast time.

However, there is a need for further studies to investigate the changes that may influence the meat's objective characteristics to confirm it.

### Use of Peracetic Acid As an Antimicrobial in Chicken

**Figure 4:**
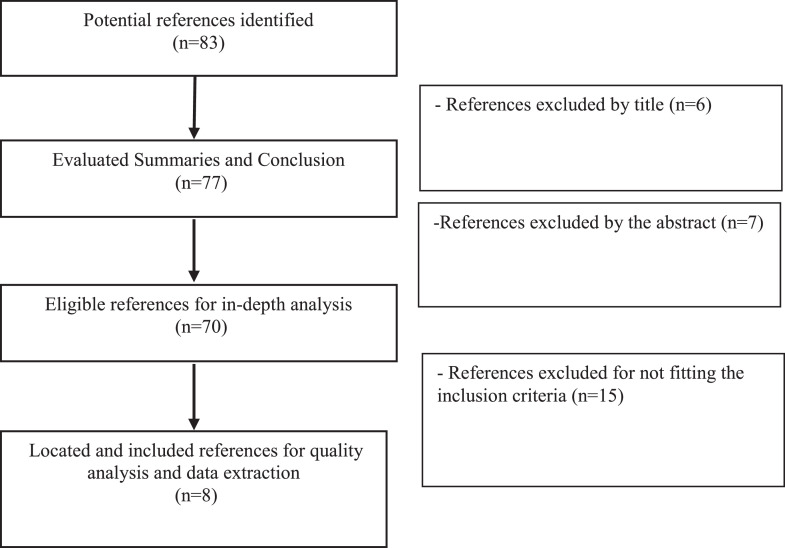
Flow diagram representing the systematic review process results.

Figure 4 represents the steps of the systematic review process summarized in 63 references with pertinent data related to the poultry process and the use of the antimicrobial PAA.

The studies found to support this review were sufficient for structuring the bibliographic information and detailing this information, for example, type of bacteria analyzed, the treatment applied with its controlled process parameters (immersion, spraying, chemical concentration, and application time), among others. In this way, the diagram contributes to the selection of key articles to be prioritized.

### Experimental Summary on the Effectiveness of PAA and Other Antimicrobials

Alongside PAA, a range of antimicrobials is being researched for decontamination in poultry meat.

Table 3 presents research studies that address the effectiveness of these antimicrobials and their comparison with PAA. The addressed antimicrobial was caprylic acid (**CA**), chlorine, cetylpyridinium chloride, combination of sulfuric acid and sodium sulfate (Amplon), use of specific phages, lauric arginate, and lysozyme.

**Table 3:** Comparison of PAA treatment effectiveness with other antimicrobials for decontamination in chicken meat.

Reference	Bacteria	Sample	Environment	Application	Treatment	Reduction (log10 UFC/g)
Ae Kim et al. (2017)	Total mesophil count	Chicken carcass	After chiller	Immersion	750 ppm PAA/15 s	4.08
Zhang et al. (2019)	*Campylobacter jejuni*	Chicken Thigh	Laboratory	Immersion	0.60% CPC/10 s	≅3.7
Zhang et al. (2019)	*Campylobacter jejuni*	Chicken Thigh	Laboratory	Immersion	0.60% CPC/20 s	≅3.7
Zhang et al. (2019)	*Campylobacter jejuni*	Chicken Thigh	Laboratory	Immersion	0.60% CPC/30 s	≅3.7
Zhang et al. (2019)	*Campylobacter jejuni*	Chicken Thigh	Laboratory	Immersion	700 ppm PAA /10 s	3.50
Zhang et al. (2019)	*Salmonella Typhimurium*	Chicken Thigh	Laboratory	Immersion	0.60% CPC/30 s	≅3.5
Zhang et al. (2019)	*Campylobacter jejuni*	Chicken Thigh	Laboratory	Immersion	0.35% CPC/10 s	≅3.5
Zhang et al. (2019)	*Campylobacter jejuni*	Chicken Thigh	Laboratory	Immersion	0.35% CPC/20 s	≅3.5
Zhang et al. (2019)	*Campylobacter jejuni*	Chicken Thigh	Laboratory	Immersion	0.35% CPC/30 s	≅3.5
Ae Kim et al. (2017)	*Campylobacter*	Chicken carcass	Before evisceration	Aspersion	Amplon (pH 1.3)	3.25
Kataria et al. (2020)	*Campylobacter coli*	Chicken Wing	Laboratory	Immersion	500 ppm PAA/10 s	≅2.5
Kataria et al. (2020)	*Campylobacter coli*	Chicken Wing	Laboratory	Immersion	500 ppm PAA/60 min	≅2.5
Zhang et al. (2019)	*Salmonella Typhimurium*	Chicken Thigh	Laboratory	Immersion	0.60% CPC/20 s	≅2.5
Zhang et al. (2019)	*Campylobacter jejuni*	Chicken Thigh	Laboratory	Immersion	1,000 ppm PAA/10 s	≅2.5
Zhang et al. (2019)	*Campylobacter jejuni*	Chicken Thigh	Laboratory	Immersion	1,000 ppm PAA/20 s	≅2.5
Zhang et al. (2019)	*Campylobacter jejuni*	Chicken Thigh	Laboratory	Immersion	1,000 ppm PAA/30 s	≅2.5
Manjankattil et al. (2021)	*Salmonella Heidelberg*	Chicken Thigh	Laboratory	Immersion	1% CA + 500 ppm de PAA	2.34
Zhang et al. (2019)	*Campylobacter jejuni*	Chicken Thigh	Laboratory	Immersion	700 ppm PAA/20 s	2.30
Ae Kim et al. (2017)	*Campylobacter*	Chicken carcass	After chiller	Immersion	750 ppm PAA/15 s	2.23
Sukumaran et al. (2015)	*Salmonella*	Chicken Skin	After chiller	Immersion	400 ppm PAA+ Phage/20 s	2.2–2.5
Sukumaran et al. (2015)	*Salmonella*	Chicken Skin	After chiller	Immersion	50 ppm PAA + Fago/20 s	2.2
Nagel et al. (2013)	*Salmonella Typhimurium*	Chicken carcass	After chiller	Immersion	1,000 ppm PAA/20 s	2.14
Nagel et al. (2013)	*Campylobacter jejuni*	Chicken carcass	After chiller	Immersion	1,000 ppm PAA/20 s	2.03
Nagel et al. (2013)	*Salmonella Typhimurium*	Chicken carcass	After chiller	Immersion	400 ppm PAA/20 s	2.02
Kataria et al. (2020)	*Campylobacter coli*	Chicken Wing	Laboratory	Immersion	50 ppm PAA/10 s	≅2
Kataria et al. (2020)	*Campylobacter coli*	Chicken Wing	Laboratory	Immersion	50 ppm PAA/60 min	≅2
Zhang et al. (2019)	*Salmonella Typhimurium*	Chicken Thigh	Laboratory	Immersion	700 ppm PAA/10 s	≅2
Zhang et al. (2019)	*Salmonella Typhimurium*	Chicken Thigh	Laboratory	Immersion	700 ppm PAA/20 s	≅2
Zhang et al. (2019)	*Salmonella Typhimurium*	Chicken Thigh	Laboratory	Immersion	700 ppm PAA/30 s	≅2
Zhang et al. (2019)	*Salmonella Typhimurium*	Chicken Thigh	Laboratory	Immersion	1,000 ppm PAA/10 s	≅2
Zhang et al. (2019)	*Salmonella Typhimurium*	Chicken Thigh	Laboratory	Immersion	1,000 ppm PAA/20 s	≅2
Zhang et al. (2019)	*Salmonella Typhimurium*	Chicken Thigh	Laboratory	Immersion	1,000 ppm PAA/30 s	≅2
Zhang et al. (2019)	*Campylobacter jejuni*	Chicken Thigh	Laboratory	Immersion	700 ppm PAA/30 s	2.00
Nagel et al. (2013)	*Campylobacter jejuni*	Chicken carcass	After chiller	Immersion	400 ppm PAA/20 s	1.93
Kumar et al. (2020)	*Salmonella Typhimurium*	Chicken Breast	Laboratory	Immersion	1,000 ppm PAA/30 s	1.92
Kumar et al. (2020)	*Campylobacter coli*	Chicken Breast	Laboratory	Immersion	1,000 ppm PAA/30 s	1.87
Kataria et al. (2020)	*Salmonella Typhimurium*	Chicken Wing	Laboratory	Immersion	500 ppm PAA/60 min	≅1.8
Kumar et al. (2020)	*Salmonella Typhimurium*	Chicken Breast	Laboratory	Immersion	500 ppm PAA/30 s	1.77
Sukumaran et al. (2015)	*Salmonella*	Chicken Skin	After chiller	Immersion	400 ppm PAA/20 s	1.7
Sukumaran et al. (2015)	*Salmonella*	Chicken Skin	After chiller	Immersion	Chlorine 30 ppm + Phage/20 s	1.7
Kumar et al. (2020)	*Campylobacter coli*	Chicken Breast	Laboratory	Immersion	500 ppm PAA/30 s	1.68
Zhang et al. (2019)	*Salmonella Typhimurium*	Chicken Thigh	Laboratory	Immersion	0.35% CPC/10 s	≅1.6
Zhang et al. (2019)	*Salmonella Typhimurium*	Chicken Thigh	Laboratory	Immersion	0.35% CPC/20 s	≅1.6
Zhang et al. (2019)	*Salmonella Typhimurium*	Chicken Thigh	Laboratory	Immersion	0.35% CPC/30 s	≅1.6
Zhang et al. (2019)	*Salmonella Typhimurium*	Chicken Thigh	Laboratory	Immersion	0.60% CPC/10 s	≅1.6
Manjankattil et al. (2021)	*Salmonella Heidelberg*	Chicken Breast	Laboratory	Immersion	1% CA + 1,200 ppm de PAA	1.57
Ae Kim et al. (2017)	*Campylobacter*	Chicken carcass	After chiller	Immersion	Amplon (pH 1.4/15 s)	1.53
Manjankattil et al. (2021)	*Salmonella Heidelberg*	Chicken Thigh	Laboratory	Immersion	0.5% CA + 500 ppm de PAA	1.52
Kumar et al. (2020)	*Salmonella Typhimurium*	Chicken Breast	Laboratory	Aspersion	1,000 ppm PAA/10 s	1.45
Manjankattil et al. (2021)	*Salmonella Heidelberg*	Chicken Breast	Laboratory	Immersion	2% CA	1.41
Kumar et al. (2020)	*Salmonella Typhimurium*	Chicken Breast	Laboratory	Immersion	250 ppm PAA/30 s	1.33
Kataria et al. (2020)	*Salmonella Typhimurium*	Chicken Wing	Laboratory	Immersion	50 ppm PAA/60 min	≅1.3
Bauermeister et al. (2008)	*Salmonella Typhimurium*	Chicken carcass	Chiller	Immersion	200 ppm PAA/10 min	1.30
Manjankattil et al. (2021)	*Salmonella Heidelberg*	Chicken Breast	Laboratory	Immersion	1,200 ppm PAA/2 min	1.25
Manjankattil et al. (2021)	*Salmonella Heidelberg*	Chicken Breast	Laboratory	Immersion	2% CA + 1,200 ppm de PAA	1.25
Kumar et al. (2020)	*Campylobacter coli*	Chicken Breast	Laboratory	Aspersion	1,000 ppm PAA/10 s	1.24
Kumar et al. (2020)	*Salmonella Typhimurium*	Chicken Breast	Laboratory	Aspersion	500 ppm PAA/10 s	1.23
Kumar et al. (2020)	*Campylobacter coli*	Chicken Breast	Laboratory	Immersion	250 ppm PAA/30 s	1.23
Sukumaran et al. (2015)	*Salmonella*	Chicken Skin	After chiller	Immersion	CPC 0.2% + Fago/20 s	1.2–1.3
Bauermeister et al. (2008)	*Salmonella Typhimurium*	Chicken carcass	Chiller	Immersion	100 ppm PAA/10 min	1.20
Manjankattil et al. (2021)	*Salmonella Heidelberg*	Chicken Breast	Laboratory	Immersion	1% CA	1.19
Ae Kim et al. (2017)	*Campylobacter*	Chicken carcass	Chiller Simulator	Immersion	Amplon (pH 1.4/15 s)	1.15
Kumar et al. (2020)	*Salmonella Typhimurium*	Chicken Breast	Laboratory	Immersion	100 ppm PAA/30 s	1.01
Kumar et al. (2020)	*Salmonella Typhimurium*	Chicken Breast	Laboratory	Aspersion	250 ppm PAA/10 s	1.01
Kataria et al. (2020)	*Salmonella Typhimurium*	Chicken Wing	Laboratory	Immersion	500 ppm PAA/10 s	≅1
Sukumaran et al. (2015)	*Salmonella*	Chicken Skin	After chiller	Immersion	LAE 200 PPM + Fago/20 s	0.8–1
Manjankattil et al. (2021)	*Salmonella Heidelberg*	Chicken Thigh	Laboratory	Immersion	1% CA	0.98
Bauermeister et al. (2008)	*Salmonella Typhimurium*	Chicken carcass	Chiller	Immersion	25 ppm PAA/10 min	0.90
Kumar et al. (2020)	*Campylobacter coli*	Chicken Breast	Laboratory	Aspersion	500 ppm PAA/10 s	0.85
Nagel et al. (2013)	*Salmonella Typhimurium*	Chicken carcass	After chiller	Immersion	40 ppm chlorine/20 s	≅0.8
Nagel et al. (2013)	*Salmonella Typhimurium*	Chicken carcass	After chiller	Immersion	5,000 ppm Lisozima/20 s	≅0.8
Bauermeister et al. (2008)	*Campylobacter jejuni*	Chicken carcass	Chiller	Immersion	200 ppm PAA/10 min	0.80
Kumar et al. (2020)	*Campylobacter coli*	Chicken Breast	Laboratory	Immersion	100 ppm PAA/30 s	0.78
Kumar et al. (2020)	*Campylobacter coli*	Chicken Breast	Laboratory	Aspersion	250 ppm PAA/10 s	0.74
Manjankattil et al. (2021)	*Salmonella Heidelberg*	Chicken Thigh	Laboratory	Immersion	0.5% CA	0.70
Nagel et al. (2013)	*Salmonella Typhimurium*	Chicken carcass	After chiller	Immersion	40 ppm chlorine/20 s	≅0/7
Nagel et al. (2013)	*Campylobacter jejuni*	Chicken carcass	After chiller	Immersion	40 ppm chlorine/20 s	≅0.7
Nagel et al. (2013)	*Campylobacter jejuni*	Chicken carcass	After chiller	Immersion	5,000 ppm Lisozima/20 s	≅0.7
Kumar et al. (2020)	*Salmonella Typhimurium*	Chicken Breast	Laboratory	Aspersion	100 ppm PAA/10 s	0.65
Sukumaran et al. (2015)	*Salmonella*	Chicken Skin	After chiller	Immersion	CPC 0.2%/20 s	0.6–0.7
Nagel et al. (2013)	*Salmonella Typhimurium*	Chicken carcass	After chiller	Immersion	Water	≅0.6
Nagel et al. (2013)	*Campylobacter jejuni*	Chicken carcass	After chiller	Immersion	40 ppm chlorine/20 s	≅0.6
Kataria et al. (2020)	*Salmonella Typhimurium*	Chicken Wing	Laboratory	Immersion	50 ppm PAA/10 s	≅0.57
Sukumaran et al. (2015)	*Salmonella*	Chicken Skin	After chiller	Immersion	Chlorine 30 ppm/20 s	0.5–0.6
Sukumaran et al. (2015)	*Salmonella*	Chicken Skin	After chiller	Immersion	LAE 200 PPM/20 s	0.5–0.6
Zhang et al. (2019)	*Salmonella Typhimurium*	Chicken Thigh	Laboratory	Immersion	0.003% Chlorine/10 s	≅0.5
Zhang et al. (2019)	*Campylobacter jejuni*	Chicken Thigh	Laboratory	Immersion	0.003% Chlorine/10 s	≅0.5
Zhang et al. (2019)	*Campylobacter jejuni*	Chicken Thigh	Laboratory	Immersion	0.003% Chlorine/20 s	≅0.5
Zhang et al. (2019)	*Campylobacter jejuni*	Chicken Thigh	Laboratory	Immersion	0.003% Chlorine/30 s	≅0.5
Ae Kim et al. (2017)	Total mesophil count	Chicken carcass	Chiller Simulator	Immersion	Amplon (pH 1.4/15 s)	≅0.5
Nagel et al. (2013)	*Campylobacter jejuni*	Chicken carcass	After chiller	Immersion	Water	≅0.5
Sukumaran et al. (2015)	*Salmonella*	Chicken Skin	After chiller	Immersion	50 ppm PAA	0.4–0.6
Ae Kim et al. (2017)	Total mesophil count	Chicken carcass	Before evisceration	Aspersion	Amplon (pH 1.3)	≅0.4
Bauermeister et al. (2008)	*Campylobacter jejuni*	Chicken carcass	Chiller	Immersion	25 ppm PAA/10 min	0.40
Sukumaran et al. (2015)	*Salmonella Typhimurium*	Chicken Thigh	Laboratory	Immersion	0.003% Chlorine/20 s	≅0.3
Bauermeister et al. (2008)	*Campylobacter jejuni*	Chicken carcass	Chiller	Immersion	100 ppm PAA/10 min	0.30
Kumar et al. (2020)	*Campylobacter coli*	Chicken Breast	Laboratory	Aspersion	100 ppm PAA/10 s	0.28
Sukumaran et al. (2015)	*Salmonella Typhimurium*	Chicken Thigh	Laboratory	Immersion	0.003% Chlorine/30 s	≅0.2
Manjankattil et al. (2021)	*Salmonella Heidelberg*	Chicken Thigh	Laboratory	Immersion	500 ppm PAA/2 min	NG
Ae Kim et al. (2017)	Total mesophil count	Chicken carcass	After chiller	Immersion	Amplon (pH 1.4/15 s)	NG
Ae Kim et al. (2017)	*Salmonella*	Chicken carcass	After chiller	Immersion	750 ppm PAA/15 s	Qualitative analysis
Ae Kim et al. (2017)	*Salmonella*	Chicken carcass	Before evisceration	Aspersion	Amplon (pH 1.3)	Qualitative analysis
Ae Kim et al. (2017)	*Salmonella*	Chicken carcass	Chiller Simulator	Immersion	Amplon (pH 1.4/15 s)	Qualitative analysis
Ae Kim et al. (2017)	*Salmonella*	Chicken carcass	After chiller	Immersion	Amplon (pH 1.4/15 s)	Qualitative analysis

Key1: CA: caprylic acid; PC: positive control; NG: no grow detected; CPC: chloride of cetylpyridinium; LAE: arginate lauric; PAA: peracetic acid.

Most of the studies focused on relevant bacteria in the poultry process, such as *Salmonella Heidelberg, Salmonella Typhimurium, Campteriaylobacter jejuni, Campylobacter coli*, as well as indicator bacteria and total mesophil count.

Different cuts were evaluated in the studies, among them chicken breast and cuts with skin, thigh, wing, skin, and chicken carcass. The range of cuts allowed a detailed analysis of the antimicrobials effectiveness in the worst case scenario, because the chicken wing and thigh cuts are completely covered with the skin and can provide protection to microorganisms and prevent antimicrobial exposure (Kataria et al., 2020).

Most of the studies (80% of them) covered in the research were conducted in laboratory settings, with the meat matrix inoculated with a cocktail of resistant strains, treated with the antimicrobial under study, and then evaluated for log reduction of the bacteria. It is worth noting that high inoculations as in the case of laboratory scale studies, in general, do not reflect the reality of a production process, however, high inoculations supposed that if the antibacterial substance is effective against some bacteria in high contamination log it will be effective when the contamination is low. The reduction found by studies using PAA alone or in combination with another antimicrobial, ranged from 0.65 to 4.08 log CFU/g where 85% of the results, showed reduction above 1 log CFU/g, which is prominently expressive.

The way of applying antimicrobials that has been most addressed in the studies is the immersion of chicken cuts or carcasses for a controlled time and concentration, the longer the immersion time, the lower the concentration used.

Kumar et al. (2020) evaluated both immersion and spray methods with chicken breast fillets treated with different concentration of PAA. The study highlighted that all levels of PAA reduced log of *Salmonella* reaching a 1.45 log CFU/g reduction in the sprinkling process when the highest concentration (1,000 ppm) was used and 1.92 log CFU/g reduction in the cut immersion process, also at the highest concentration. It can be observed that for both addressed methods, the increase in logarithmic reduction occurred as the concentration of the antimicrobial solution increased. The study also showed satisfactory results for the reduction of *Campylobacter coli*, where at the highest concentration a 1.87 log CFU/g and 1.24 log CFU/g reduction were obtained for the immersion and aspersion processes respectively. Thus, the application of the PAA treatment by immersion was more effective in reducing *Salmonella* and *Campylobacter* populations in chicken breast fillets when compared to the spray method; however, the results found with the spray method of application were also significant.

An interesting approach to antimicrobial application methods was the evaluation of chicken carcasses after the scalding step, before entering the evisceration process. The study in focus evaluated the effectiveness of the Amplon antimicrobial by the carcass sprinkling method and assessed the total mesophil count (APCs) and *Campylobacter* counts. The reduction of APCs on chicken carcasses did not show significant differences in the bacteria recovered (*P* > 0.05) between the control and treated groups, indicating that spraying Amplon did not affect the APC populations on chicken carcasses; but, regarding *Campylobacter*, spraying with the antimicrobial resulted in a log reduction of 3.25 CFU/g (Ae Kim et al., 2017). This application strategy after the scalding stage contributes to the reduction of the microbial load throughout the production process and not only at the end of the process, since the stage before the treatment (scalding) may result in the initial fixation of bacteria on the skin, carrying the contamination along the subsequent stages of processing (McBride et al., 1980).

Manjankattil et al. (2021) investigated the effectiveness of 2 compounds, CA and PAA, alone or in combination, against *Salmonella Heidelberg* in the breasts and thighs of broilers. The most effective method was PAA in combination (1% CA + 500 ppm PAA) achieving a 2.34 log CFU/g reduction.

Kataria et al. (2020) evaluated the effectiveness of PPA on chicken wing cuts treated with 50 and 500 ppm PPA for 10 s and 60 min of immersion. The survey showed better results for *Campylobacter* reduction: at the highest concentration for both 10 s and 60 min the study found an approximately 2.5 log CFU/g reduction in *Campylobacter* count.

The effectiveness of different treatments, 0.003% chlorine, 700 ppm PAA, 1,000 ppm PAA, 0.35% cetylpyridinium chloride (**CPC**) and 0.6% CPC for 10, 20, and 30 s contact time, was evaluated against *Salmonella Typhimurium* and *Campylobacter jejuni*. All treatments were evaluated by immersion method. PAA treatment reduced *Salmonella* by approximately 2 log CFU/g for both concentrations (700 and 1,000 ppm) and CPC treatment (0.35%) also showed a satisfactory reduction in *Salmonella* approximately 1.6 log CFU/ g for all times evaluated in the study. For *C. jejuni* the results treated with PAA and CPC were more effective when compared to the *Salmonella* results, showing at their best, reductions of approximately 3.5 log CFU/g when subjected to PAA treatment and approximately 3.7 log CFU/g when treated with CPC. Regarding chlorine, no significant (*P* > 0.05) reductions in microbial counts was observed (Zhang et al., 2019).

Bauermeister et al. (2008) conducted a comparative evaluation of chicken carcasses treated with different levels of PAA to reduce *Salmonella* and *Campylobacter* compared to chicken carcasses treated with chlorine in the chiller system of a commercial processing facility. The study showed that all levels of PAA reduced CFU/sample of *Salmonella* and *Campylobacter* significantly (*P* ≤ 0.05) more than chlorine.

Concerning postchiller applications, another paper discussed the effectiveness of postchiller water treatments consisting of 40 ppm total chlorine, 400 ppm or 1,000 ppm PAA, and 1,000 ppm or 5,000 ppm lysozyme against *Salmonella* and *Campylobacter* spp. Comparing the antimicrobial efficacy of the various antimicrobials, PAA showed the best reduction (*P* ≤ 0.05) in *S. Typhimurium* and *Campylobacter jejuni* in chicken carcasses. The best logarithmic reductions were 2.14 and 2.03 log CFU/mL for the treatments using the highest concentration (1,000 ppm), which corroborates with the studies presented in Table 3, demonstrating that raising the concentration of PAA for the immersion system corroborates with the logarithmic reduction (Nagel et al., 2013).

An interesting trend is the combination of PAA and a second antimicrobial agent to improve the effectiveness of the chemical antimicrobial. Sukumaran et al. (2015) evaluated chicken skin samples inoculated with a cocktail of *Salmonella Typhimurium, S. Heidelberg*, and *S. Enteritidis* immersed in an antimicrobial solution of different chemical antimicrobials (LAE lauric arginate, CPC, PAA, or chlorine) for 20 s and then were surface treated with a *Salmonella*-specific bacteriophage (phage) solution applied via spray. When the phage was applied sequentially with chemical antimicrobials, all treatments resulted in significant reductions of *Salmonella*. The application of PAA (400 ppm) followed by the spray application of the phage resulted in the greatest *Salmonella* reduction of 2.2 to 2.5 log CFU/cm². In conclusion, the greatest reductions in *Salmonella* counts were achieved in chicken skin by sequential application of chemical antimicrobials followed by phage spray, this sequential application (chemical antimicrobial + phage) may provide additional obstacles for pathogen reduction in carcasses or poultry parts.

PAA is the most popular antimicrobial in poultry farming, replacing chlorine, and other compounds. Cano et al. (2021) compared in their review research, the effectiveness of PAA vs. other antimicrobials for the decontamination of raw poultry carcasses and parts. For this purpose, the author reviewed 26 articles that compared PAA with more than 20 different antimicrobials, using different applications (spray or immersion), concentrations, and exposure times. The compounds often found as a comparator were chlorine, lactic acid, and cetylpyridinium chloride. The pathogens involved in the studies were mostly *Salmonella* and *Campylobacter*. The review also corroborates the efficacy of the antimicrobial action of PAA for pathogen control.

## FINAL CONSIDERATIONS

PAA used as an antimicrobial intervention for poultry, either alone or when combined with other antimicrobial compounds, is consistently effective in reducing major pathogens (*Salmonella* and *Campylobacter*) and microbial loads in the course of the process, and is a unique strategy as technological barriers in poultry processes.

The method of immersing the cuts after the cooling system has been the most widely used in the published literature, which ensures the safety of the final product. The treatment at the end of the production process avoids possible recontamination of the product through cross contamination. Nonetheless, application at critical steps of the poultry slaughter process, such as after scalding and during evisceration, is a unique strategy to contain microbial spread, reducing the initial load of incoming bacteria and stabilizing control throughout the process.

More research is needed and should be conducted addressing studies with the strain found in the plant alongside its actual initial count. Most studies approach research by inoculating the meat matrix with a cocktail of resistant strains, applying treatment with the antimicrobial under investigation, and then evaluating the logarithmic reduction of the bacteria. It is important to emphasize that the reality of the slaughter process may bring different variables: strains that may be more resistant than the inoculation strains, initial microbial counts, and variations in the application of antimicrobials in the slaughter line. In general, the scenario inside a laboratory does not reflect the reality of a production process

In regards to the technological effects of the PAA antimicrobial on poultry meat, further studies are needed to further investigate the modifications that may influence the subjective characteristics of the meat as well as consumer perception.

Finally, to ensure the risk assessment of the HEDP stabilizer corroborating with the safety of the EAP, studies that present HEDP residuals for long-term immersion bath applications in poultry carcasses, poultry meat, and poultry meat products are of paramount importance to enable the validation of an effective method that can determine and quantify this residual. Due to the basic stoichiometry, phosphorus functions as an indicator of the presence of HEDP and perhaps, could be a pathway for the development of a method to be validated. Thus, future studies that present this approach and that corroborate the safety of the stabilizer in the formulation of PAA will contribute to the approval of meat sanitary regulations in several countries.
